# Parent-Infant Interaction during the First Year of Life in Infants at High Risk for Cerebral Palsy: A Systematic Review of the Literature

**DOI:** 10.1155/2019/5759694

**Published:** 2019-04-15

**Authors:** F. Festante, C. Antonelli, O. Chorna, G. Corsi, A. Guzzetta

**Affiliations:** ^1^Department of Developmental Neuroscience, IRCCS Fondazione Stella Maris, Pisa, Italy; ^2^Department of Clinical and Experimental Medicine, University of Pisa, Pisa, Italy

## Abstract

**Introduction:**

Perinatal adverse events put neonates at high risk for short and long-term disabilities, including cerebral palsy (CP). The most recent guidelines about early intervention in infants with brain damage have emphasized the importance of family involvement from the very first phases of development. Early parent-infant interactions are pivotal in promoting infant cognitive and social developmental trajectories. However, little is known about the extent to which severe adverse perinatal events can affect the quality of early parent-infant interactions.

**Patients and Methods:**

We systematically searched five databases (PubMed, PsycINFO, EMBASE, CINAHL, and Cochrane Library) for the publications assessing parent-infant interactions in infants at high neurological risk within 1 year of age. Articles were selected if they involved direct comparison between high-risk populations and healthy controls or low-risk populations, and if quantitative or semiquantitative tools were used to assess the parent-infant interaction. Measures of parent-infant interaction included infant interactive behaviors, parental interactive behaviors, and dyadic interactive patterns.

**Results:**

The search yielded 18 publications that met the inclusion criteria. The articles represent a high level of heterogeneity in terms of infant neurological risk, infant age, and tools assessing interactive behaviors. Both infant and maternal behaviors within the investigated interactive exchanges were reported to be compromised, leading to subsequent overall impairment of the dyadic patterns.

**Conclusion:**

While the studies reviewed here provide general and important information, the review did not yield a clear picture of early dyadic interactions in high-risk infant populations. Further observational studies are warranted in order to provide a more accurate knowledge of the early dyadic exchanges between infants at high neurological risk and their parents, as they might provide a critical opportunity for early family centered habilitative interventions.

## 1. Introduction

The role of parent-infant interaction during early development has been studied extensively in the past decades. Newborn's brain is known to be prone to interactive exchanges at birth or even before [1, 2]. Neonatal imitative processes, occurring from the very first hours of life [2], represent the first signs of reciprocity between parents and infants that, within the first months of life, evolve towards actual *protoconversations*, characterized by reciprocal multimodal exchanges and rhythmic vocal, facial, and gesture imitations [3–5]. Murray and colleagues have recently suggested the existence of a functional architecture of mother-infant engagements, active from the very first weeks of life and apt to support the development of infant intersubjective skills [6]. Authors reported that the occurrence of mirroring or marking maternal responses to infant social expressions predicts the increase of such infant behaviors over time. More importantly, they stressed the importance of contingency more than frequency of maternal responses, thus suggesting that infants are able to capitalize on relatively limited exposure to specific parental behaviors, already at very early developmental stages.

Primary dyadic interactions support infants' cognitive, motor, and social skills maturation [7, 8]. Studies in typically developing infants have widely demonstrated that the quality of early dyadic interactions can strongly influence later infants' developmental outcomes [9–12]. For instance, Feldman and Greenbaum [10] reported that maternal affective attunement and dyadic synchrony within the interaction of 3-month-old infants and their mothers were predictive of infants' quality of play, verbal IQ, and regulation capacity at 2 years of age. The contingency within interactive exchanges at 3 months of age has been reported to be a precursor of infants' attachment style at 1 year [13], while maternal sensitivity to infant distress has been described as a predictor of a secure attachment [14]. Accordingly, studies focusing on clinical populations showed that the occurrence of either parents adverse conditions (e.g., maternal depression, anxiety or early traumatic experiences, and poor socioeconomic family) or infant pathological conditions (e.g., preterm birth, autism, and cleft lip palate) can be associated with poor infant affective, social, and cognitive outcomes, likely due, at least partly, to a disruption of the quality of early dyadic interactions [7, 15–17].

So far, little attention has been given to the quality of early dyadic interaction in infants at high risk of neuromotor disabilities, and more specifically of cerebral palsy (CP), as studies on term infants with brain damage are very scarce, while the majority of the studies on preterm infants have focused on low-risk prematurity [18, 19].

CP is the most common physical disability in childhood [20]. Despite the progressive improvements in perinatal and neonatal care, extreme prematurity as well as perinatal insults are still associated with major neonatal morbidities with long-term sequelae such as neurodevelopmental delay, neurosensory disorders, and cerebral palsy [21–25]. More specifically, in populations of very or extremely premature infants or of full-term infants with a history of perinatal asphyxia, the prevalence rate of CP is still consistently above 10% in high-income countries [26–30]. Scientific evidence is rapidly growing in support of the importance of an early diagnosis of CP for the improvement of long-term outcomes [31]. This is essential for a prompt referral to early intervention programs aimed at promoting and maximizing neuroplasticity, minimizing further medical complications [31], and providing emotional support for parents [32–34].

The most recent guidelines about early intervention in infants with brain damage have greatly emphasized the importance of family involvement from the very first phases of development [35]. Indeed, review studies in infants at very high risk of CP indicate that early interventions focusing on parents' empowerment and supporting early parent–infant relationships may have a greater impact on later cognitive and neuromotor outcomes compared to those with an exclusive focus on infant functional impairment [36, 37]. A deep knowledge of general and detailed aspects of parent-infant interaction in populations at high risk of CP would be therefore essential to inform new strategies for early clinical support in both infants and parents. Unfortunately very little is known about how the parent-infant dyad is affected by the occurrence of severe perinatal events. In order to contribute to bridging this knowledge gap, we systematically reviewed the existing literature on early dyadic interactions between parents and infants at high risk of neurological impairments. The main objective of this paper was to review the current knowledge on the influence of severe adverse perinatal events on the quality of early parent-infant interaction, focusing on infant behavior, parental behavior, and dyadic interactive patterns. We specifically focused on papers evaluating dyadic interactions occurring within the first year of life, as the optimal time window of the emergence and early development of infant and parental interactive patterns.

## 2. Methods

### 2.1. Literature Search and Selection of Studies

A systematic literature search was performed in February 2018, through the following electronic databases: PubMed/MEDLINE, PsycINFO, EMBASE (OVID), CINAHL, and Cochrane Library. No publication date limits were applied to the searches.

The following search strategy, including both MeSH headings and keywords, was used: (Parent-child relations (Mesh) OR Mother-Child OR Father-Child OR Parent-Child OR Mother-Infant OR Father-infant OR Parent-infant) AND (Interaction^∗^ OR Relation^∗^ OR Attachment^∗^ OR Bond^∗^ OR Intersubjectiv^∗^ OR Transact^∗^) AND (Brain injuries (Mesh) OR Brain damage^∗^ OR Brain Injury^∗^ OR Brain lesion^∗^ OR Brain malformation OR Asphyxia OR Hypoxia OR Ischemia OR Encephalopathy OR Hypoxic Ischemic Encephalopathy OR Cerebral stroke OR Leukomalacia OR Hemorrhage OR Haemorrhage OR High-risk) AND (Infant (Mesh) OR Infant OR Newborn^∗^ OR Neonate^∗^ OR baby OR Preterm OR Premature).

The list of records was first checked for duplicates using EndNote (EndNote X8.2, bld 13302). Subsequently, two authors (FF and CA) independently reviewed the remaining records for suitability by title and abstract. Finally, full-text articles addressing the topic of interest were screened in order to exclude those not meeting inclusion criteria. Secondary searches involved checking of publication reference lists and manual searches of relevant journals.

Agreement for articles inclusion was reached upon discussion between authors (FF, CA, and AG).

### 2.2. Inclusion Criteria

Article selection was restricted to peer-reviewed research articles published in English and to human studies. Articles were selected if they met all of the following criteria: (1) the study involved direct comparison between at least one population of infants at high risk for neurological impairment and either healthy controls or low-risk populations, (2) mother-infant and/or father-infant and/or both parents-infant interactions were assessed, (3) quantitative or semiquantitative tools were used to assess the interaction, (4) the study included assessments within the first year of the infant's life.

High risk for neurological impairment was defined by one or more of the following conditions: gestational age (GA) at birth under 30 weeks, birth weight (BW) below 1500 g, perinatal asphyxia or hypoxic ischemic encephalopathy, cerebral stroke, periventricular leukomalacia, severe intraventricular hemorrhage (grade III or IV), or any type of documented brain damage occurring within the first month of life. Populations were defined as at high risk for neurological impairments if at least 50% of the participants met the above criteria.

No limitations for article inclusion were applied to parent-infant interaction assessment methods, which could include feeding sessions, face-to-face interactions, and free or structured play sessions, either toy-centered or non-toy-centered. Similarly, early interaction scoring modalities including scoring scales, manuals, or checklists were included provided that a clear description of the analyzed parental and/or infant interactive dimensions (e.g., maternal intrusiveness, infant engagement, and dyadic synchrony) were reported.

### 2.3. Data Extraction

Descriptive information of all included articles was systematically extracted and gathered in an electronic database. These included authors, year, study design, sample size, inclusion and exclusion criteria for clinical and/or control samples, age of infants at the time/s of the parent-infant interaction assessment, assessment methods (e.g., place, duration), scoring modalities (e.g., behavioral annotation), and main findings relative to the early parent-infant interaction. Additional parental, neonatal, or developmental outcome measures and any other results relevant to the current report were scored and gathered, if reported. The quality of the included studies was assessed by using the National Heart, Lung, and Blood Institute (NHLBI) Quality Assessment Tool for Case-Control Studies [38], which was chosen based on the study design of the included articles. Two authors (OC and FF) independently evaluated the items of the tool as “yes,” “no,” “not applicable,” “cannot determine,” or “not reported.” The comprehensive evaluation of all items was then used to rate the global quality of each study as “good,” “fair,” or “poor.”

### 2.4. Measures of Parent-Infant Interaction

Measures of parent-infant interaction were grouped into three categories. (i) Infant interactive behaviors included all behaviors originated by the infant as either initiations or responses within the assessed interactions, such as activity (e.g., movements, vocalizations, or expressive language) and engagement (e.g., facial expressions or eye contact). (ii) Parental interactive behaviors included all dimensions originated by the parent as either initiations or responses within the assessed interactions, such as sensitivity, vigilance, intrusiveness, and emotional involvement (e.g., kinesthetic or proximal stimulation, smiling, vocalizations, time spent looking at infant, and proximity to infant). (iii) Dyadic interactive patterns included all behaviors of the dyad observed as a single entity within the assessed interactions, such as synchrony, reciprocity, and coregulation (e.g., timing, rhythmicity, and fluency of interactive exchanges).

## 3. Results

**Figure 1:**
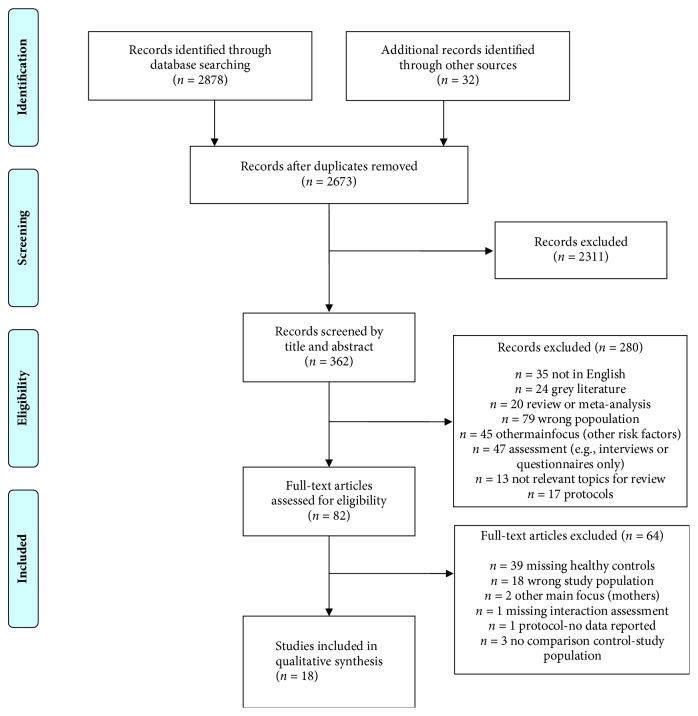
PRISMA flow chart. Flow chart of literature search and study selection.

The flow chart in Figure 1 summarizes the whole selection process and exclusion criteria at each selection step. Database and secondary searches yielded 2910 articles. After duplicates removal, titles and abstracts of 2673 articles were screened. Then, 82 full-text articles were scored, of which 18 met all predetermined inclusion criteria and were included in the present review. Overall, the quality of the studies was evaluated as fair or good with the exception of one paper whose quality was rated as poor. Details of the studies, including the quality rating, are summarized in Table 1.

### 3.1. Level of Neurological Risk of the Study Populations

All studies included preterm populations, while few of them involved mixed populations of preterm and full-term infants at high risk of neurological impairments. The severity of infant risk status was varied among the selected articles. In 8 articles, the high-risk population presented intraventricular hemorrhage (grades III–IV), periventricular leukomalacia, severe perinatal asphyxia, seizures, meningitis, or other severe medical conditions [39, 45, 46, 49–51, 55, 58], while in the remaining studies, the high-risk population included infants on the basis of intrauterine growth retardation (IUGR), birth weight, or prematurity with or without mild or moderate medical complications [41, 42, 53, 60, 62, 64, 65, 67, 69, 70].

Seven of the 18 studies explicitly excluded infants with severe brain lesions or perinatal asphyxia from their high-risk samples [50, 51, 60, 62, 67, 69, 70]; however, the resulting populations still met at least one of our inclusion criteria for high neurological risk (i.e., BW or GA) and were therefore retained in the current review.

### 3.2. Parent-Infant Interaction Assessment Modalities

The time duration for observation of early parent-infant interaction was also heterogeneous, varying from few minutes long video-recorded sessions (from 3 to 20 minutes) [39, 49, 51, 53, 58, 62, 64, 67, 69] to much longer (up to 120 minutes) live observations [41, 42, 46, 50]. A wide variety of parent-infant interaction coding modalities was used, including microanalytic coding systems [42, 45, 49, 58, 60, 70], rating scales [46, 53, 55, 62, 64, 65, 67, 69], and scoring checklists [39, 41, 51]. Only one study used a mixed coding system which included both microanalytic and global rating scores [50]. Assessment details are summarized in Table 2.

The timing at which parent-infant interaction assessments were performed was equally distributed over the first year of the infant life, with about half of the included studies collecting data within the first semester of life [39, 42, 51, 53, 58, 60, 62, 64, 67, 69] and half focusing on older infants, from 6 to 12-month-old infants [41, 45, 46, 49, 50, 55, 65, 70]. Most of the studies involved a cross-sectional design [39, 41, 45, 46, 53, 58, 60, 64, 67, 69, 70], while few of them implemented a longitudinal design, with data collection at multiple time points [42, 50, 51, 65]. Three studies [49, 55, 62] evaluated mother-infant interaction over a longer period of time, which was beyond the first year of life. For these studies, however, only assessments that occurred within the first year of life were considered in the present review.

Finally, all studies focused on mother-infant interaction. Feldman [64] was the only study that, in addition to the mother-infant interaction, also included father-infant and triadic early interactions. The authors found no differences between father-infant and mother-infant interactions; therefore, we abstain from discussing this topic further in this review.

### 3.3. Comparison of Interactive Patterns between High-Risk and Non-High-Risk Dyads prior to 6 Months

Overall, all of the included studies described compromised dyadic interactive patterns between mothers and infants who experienced adverse perinatal events, compared to those occurring within control populations. While most of the studies focused on interactive behaviors considering mother and infant as discrete interacting units, few studies analyzed the quality of early interactive exchanges from a dyadic perspective. Due to the variety of the interaction assessments and the scoring modalities utilized within the included studies, a wide range of parental and infant dimensions were analyzed. However, a categorization of common terms used within the selected publications is included for our reporting purposes (see bold text in the following section). Therefore, below, we report the findings in three sections, divided into groups of behaviors or behavioral dimensions.

#### 3.3.1. Infant Interactive Behavior

Within the first semester of life (corrected age for prematurity), infants at high risk were generally described as less active, less engaged in the interaction, and more fretful than controls. Minde and colleagues [42] reported that preterm infants were less alert and focused, as revealed by the fact that they spent less time with their eyes open during feeding sessions at one month of age, although they became more physically active by the third month of life. Davis et al. [53] described preterm infants at risk as less responsive to their caregivers compared to typically developing infants, during feeding. Schmücker et al. [58] and Feldman [64], instead, found that high-risk preterm infants were less optimally engaged in dyadic interaction with their mothers, as they showed less facial expressions and more negative engagement cues, respectively, compared to controls. Interestingly, however, Schmücker and colleagues [58] also reported that preterm infants were more vocally active and responsive than full-term infants were, thus indicating that the extent of responsiveness to the caregiver can be different depending on the communicative channel.

Two studies explored populations of full-term infants at high neurological risk, reporting abnormal infant behaviors within the parent-infant interaction [39, 51]. Specifically, Greene and colleagues [39] found that healthy infants looked significantly more at their mothers than sick infants, with sick full-term infants, corresponding to the group at highest neurological risk, having the lowest scores compared to healthy controls. Similarly, results by Schermann-Eizirik and colleagues [51] revealed that, unlike preterm born infants, full-term infants who required intensive care at birth, thus supposedly corresponding to the most impaired infants, differed from healthy full-term controls in their interactive patterns, with the first group showing significantly less interactive behaviors than the second one.

Finally, more recent studies investigating early mother-infant interactions within the first trimester of life showed no significant differences in terms of negative engagement or interactive patterns between preterm infants and full-term controls [62, 67]. Only one study [69] reported more communicative behaviors in very low birth weight (VLBW) infants compared to full-term ones.

#### 3.3.2. Parent (Mother) Interactive Behavior

Consistent with the results on infant behaviors during the first months of life, mothers of high-risk infants were described as less sensitive, more vigilant or intrusive, and less emotionally involved than mothers of healthy or low-risk infants.

At 3 months of age, Greene and colleagues [39] reported that, compared to healthy infants, high-risk infants, and specifically high-risk full-term infants, received more proximal and kinesthetic stimulation, but less distal and affective behaviors from their mothers during free play interactions. At the same infant age, other studies [62, 64, 67] reported that mothers of high-risk infants had more intrusive and less remote behaviors during face-to-face interaction than mothers of controls. Similarly, Minde and colleagues [42] reported that during feeding sessions (1 and 2 months) and play interactions (3 months), mothers of premature infants at higher risk provided more compensatory care (e.g., vocalization and face-to-face look), but less affect (e.g., smiling) to their infants compared to full-term mothers.

More inconsistent results were found about maternal sensitivity. In fact, while some studies [53, 58, 67] failed to find differences between study and control mother groups in the sensitivity dimension, others showed that mothers of high-risk infants were less sensitive than mothers of controls were [51, 60]. In particular, Schermann-Eizirik and colleagues [51] did not find differences in interactive behaviors between mothers of preterm infants, some of whom required intensive care, and mothers of healthy full-term infants. However, they observed significantly less sensitivity and less interactive involvement in mothers of high-risk full-term infants compared to mothers of healthy full-term infants at 4 and 6 months. Only one study reported enhanced sensitivity in mothers of high-risk infants, which was, however, associated with a higher level of intrusiveness [69].

Taken together, all these studies support the idea that mothers of high-risk infants are particularly focused on close monitoring and stimulating their infants rather than interacting with them in an affectionate or social manner.

#### 3.3.3. Dyadic Interactive Patterns

Among the studies included in the current review, some approached a dyadic perspective, in addition to analyzing discrete maternal and infant dimensions, and focused on compromised patterns of synchrony [60], reciprocity [64], and positive exchanges within the dyad [51]. Two studies focused on the dyadic synchrony and reciprocity in the first semester of life and revealed that dyads at risk were less synchronized than control dyads were, showing less reciprocal rhythmic and fluent exchanges [60, 64]. The study by Schermann-Eizirik and colleagues [51] revealed that high-risk dyads showed less positive exchanges compared to control dyads.

### 3.4. Comparison of Interactive Patterns between High-Risk and Non-High-Risk Dyads from 6 to 12 Months

Studies focusing on older infants evaluated more heterogeneous and difficult-to-compare interactive parameters, such as play, social, and communication skills. Consequently, also the results of these studies were more heterogeneous than were those observed during the first semester of life.

#### 3.4.1. Infant Interactive Behavior

During toy-centered play sessions, both Muller-Nix et al. [55] and Landry et al. [45] found no significant differences between the study and control groups in infant play-interactive patterns, at 6 and 12 months, respectively. Conversely, significant differences in play-strategies between 12-month-old high-risk and control infants were found by Landry et al. and by Korja et al. [49, 65]. Results by Landry and colleagues [49] revealed that high-risk infants showed, in general, less exploratory capacities compared to low-risk and healthy full-term infants and, more specifically, that high-risk infants were more dependent on mothers' structuring strategies than controls were. Similarly, Korja et al. [65] described 12-month-old preterm infants as less skilled in play, less attentive, and more apathetic, passive, and avoiding than controls during free play interactions.

A different approach was used by Farel and colleagues [46] who investigated interactive behaviors during feeding at 8 months of age and found that high-risk infants showed less clarity of cues and less responsiveness to their mothers than did controls. Finally, Smith and colleagues [50] found that high-risk infants had significantly lower expressive language abilities than controls during daily activity.

#### 3.4.2. Parent (Mother) Interactive Behavior

As far as maternal behaviors are concerned, results of studies focusing on older infants highlighted two main altered dimensions. Consistent with studies focused on younger infants, mothers of high-risk older infants seemed to be more stimulating and less sensitive toward their infants than did mothers of control infants. For example, Landry et al. [45] found more attention-directing behaviors in high-risk mothers than in mothers of controls, while Farel and colleagues [46] found that mothers of high-risk infants reached lower scores in fostering cognitive growth behaviors than control mothers did.

Less affective behaviors were found in mothers of high-risk infants by Lasky et al. [41], Muller-Nix et al. [55], and Sansavini et al. [70]. Lasky and colleagues [41] reported that mothers of preterm infants restrain their infants less during a stressful clinical examination, maybe because they were more used to this kind of procedure. Muller-Nix et al. [55] found a negative gradient of maternal sensitivity, with mother of high-risk infants being less sensitive than mothers of low-risk infants that, in turn, were less sensitive than mothers of full-term infants. The study by Sansavini and colleagues [70], instead, revealed that mothers of extremely small for gestational age infants showed lower positive affect compared to mothers of full-term infants.

Finally, two studies [50, 65] found no differences in maternal interactive behaviors between high-risk and control infant populations. However, both studies, differently from the other studies analyzing multiple discrete maternal dimensions, only reported a global score of maternal behaviors. Thus, whether significant differences would have been found, should single discrete maternal behaviors be analyzed, remains unanswered.

#### 3.4.3. Dyadic Interactive Patterns

Among the studies focusing on dyadic interactive patterns at later infants' ages, Farel and colleagues [46] showed that high-risk dyads reached significantly worse total interactive scores, during feeding at 8 months, compared to control dyads. Korja and colleagues [65] found no differences in dyadic mutuality, flatness, and disorganization and tension between 12-month-old high-risk and control infants. Finally, the study by Sansavini and colleagues [70] reported that extremely low gestational age (ELGA) dyads showed less frequent symmetrical coregulation and more frequent unilateral coregulation, specifically meaning that mothers observe, initiate, and demand doing something, while infants do not respond to them.

### 3.5. Coding Systems Used in Multiple Studies

In order to evaluate if similar patterns of mother-infant interaction could be inferred, we compared the quantitative results of those studies using the same coding systems. Only four scales were applied in more than one study (see Table 2).

The scale by Gunning et al. [68] and Murray et al. [15, 73] was used in two studies, in populations with the same characteristics and at the same age of assessment [67, 69]. Consistent results were reported in “intrusiveness” and “remoteness” dimensions, describing mothers of high-risk infants as more intrusive and less remote than mothers of controls, while inconsistent results were reported for the “sensitivity” dimension.

The NCAST scale was also used in two studies [46, 53]. The authors reported similar results in the feeding subscale, with a higher score in populations considered at high risk compared to control groups or normative data. However, investigated populations differed in clinical risk and age at the time of the mother-infant interaction assessment.

The CIB was used in other two studies [62, 64]. They both found significant differences in the “intrusiveness” dimension, with mothers of the risk group reported to be more intrusive than mothers of the control group. However, the population characteristics of the two studies and the age at the time of the mother-infant interaction assessment differed between the two studies.

Finally, two studies by Landry et al. [45, 50] used the same coding system which was developed by the authors. Comparison of the data was however not feasible, as the results in one paper [45] were only related to mother behaviors, while the results of the other [49] were only related to infant behaviors.

No further quantitative comparisons, nor meta-analysis, were feasible with the selected articles, due to the heterogeneity of the publications and because the assessment tools and the dimensions analyzed were not consistent across the reports.

## 4. Discussion

In the last thirty years, extensive research has provided evidence that early interactive exchanges are fundamental in fostering later social and cognitive development, as they steadily drive, throughout early infancy and toddlerhood, emerging infant social abilities toward intentional and more complex relational capacities [3–6, 9, 10, 71]. The occurrence of adverse perinatal events, however, negatively impacts the overall infant neurodevelopment with consequent detrimental effects also on infant social and relational dimensions [7, 8, 11, 15, 17]. The extent to which severe neonatal complications, such as the extremely preterm birth or low birth weight or the occurrence of neonatal brain insults, might affect early interactive exchanges between infants and their primary caregivers is, however, mostly uncharted. Our main objective was to review the studies that included the emerging behavioral interactive patterns of parent-infant dyads in infant populations at high neurological risk compared to control populations. We specifically focused on parent-infant interactions occurring over the first year of life, as it represents one of the most critical stages in infants' neurodevelopment and therefore is considered an optimal timeframe for early intervention on infants experiencing consequences of brain damage including developmental delays.

Most of the 18 studies resulting from our systematic search revealed that both infant and maternal behaviors within early interactions are compromised, which results, in turn, in a more general impairment of dyadic patterns. During the first six months of life, infants at high neurological risk are described as less engaged and active than control infants, which makes them less responsive social partners, unable to communicate cues that are sufficiently clear to their caregivers [47, 58, 60]. The most likely explanation of this finding is that these altered behaviors are primarily dependent on infants' neurophysiological immaturity and medical conditions, which necessarily affect their propensity to interact. This is consistent with the findings by Feldman [60] who evaluated neonatal biological rhythms and their relationship with mother-infant synchrony at 3 months of age. The author found that immature or dysregulated endogenous rhythms, due to perinatal events, limit the capacity of arousal modulation and negatively affect infant emotionality. During the same timeframe, i.e., the first semester of life, mothers of high-risk infants are more intrusive and overstimulating but, at the same time, less sensitive and affective [51, 60]. Authors have generally interpreted these behaviors as the result of major maternal concerns relative to the health status of their infants. In this view, mothers tend to be more focused on infants' caretaking while lacking emotional involvement [39, 58].

It is of interest that the abnormalities observed in infant behavior tend to persist beyond the first semester of life, with infants being less engaging in the interaction and less focused during play sessions, while the intrusive maternal behaviors observed in early interactions evolve into more controlling and attention-directing behaviors in the second semester of life [45, 49]. This has been interpreted as a process by which mothers become more conscious of their infants' compromised motor, cognitive, and communicative skills and shift from early concerns to the adoption of more suitable strategies to optimize their interactions with infants, such as directing their attention. Taken together, these results suggest that communicative patterns between mothers and infants at risk are less fluent and more disorganized compared to those typically observed in healthy populations and that the resulting quality of interaction is significantly impaired on the short and long terms.

Findings from the current review do not provide an exhaustive characterization of early mother-infant relationships in populations at very high risk of developing CP. In fact, many of the studies excluded infants with major neurological complications, thus leaving low birth weight and extreme prematurity as the primary selection criteria. While this selection approach limited the heterogeneity of the investigated samples, it clearly reduced the overall level of neurodevelopmental risk of the populations defined as at high risk. In spite of this important limitation, the available data support the concept that illness, rather than prematurity per se, gives the greatest contribution to the disruption of early infant interactive behaviors and, in turn, of maternal responses. In particular, our findings show that infants with more severe illness, either born prematurely or at term, have less optimal interactive approaches toward their mothers, as opposed to those with lower levels of neurodevelopmental risk [39, 41, 42, 45, 46, 50, 51, 58]. Similarly, maternal behaviors are directly related to infants' medical status with greater levels of infant risk associated with greater alterations of maternal interactive behaviors [42, 50, 51, 58]. In general, mothers of infants who faced major neurological complications at birth were also more depressed, distressed, and anxious, as revealed by postnatal interviews or questionnaires, and these emotional states seem to influence mothers' interactive behaviors toward a less efficient perception of their infants' cues [55, 58, 67, 69]. However, some inconsistency was found in relation to this aspect. Some other studies have found weak or no significant impact of maternal emotional states on the mother-infant interaction in high-risk populations, differently from the control population in which they seemed to play a bigger role [42, 53, 58]. Specifically, more negative emotional states were associated to poorer or more negatively affected maternal interactive patterns. Finally, few and discordant results were identified on the extent to which maternal emotional state compared to infants' risk can alter the interactive patterns [55, 58, 64, 69]. Further investigations are therefore needed to disambiguate such aspects and, more importantly, to provide deeper insights on the maternal emotional state following the occurrence of perinatal adverse events and on the extent to which they can influence maternal interactive patterns over time.

It is of interest that the studies included in this work cover a time span of over thirty years. However, only older studies focused on very high neurological risk populations, while most recent ones mainly focused on prematurity. Two main considerations can be made. First, it is plausible that since first attempts at investigating early interactions in such complex populations have not been fully successful, subsequent attempts have mainly deviated toward more homogeneous populations including only premature infants. On the one hand, this approach has led to more consistent and reliable knowledge on early interactive exchanges in premature populations. On the other hand, however, this has also left many unanswered questions about the role of neurological illness in early mother-infant interactions. Second, the survival rate of infants at high risk for neurological impairments as indicated by current guidelines was significantly lower in the past decades, while it has significantly increased following advances in perinatal and neonatal care. Currently, more than ever, there is a critical need for the prompt referral of high-risk infants to diagnostic-specific early intervention, promoting early social interactions.

It is important to underline that findings were not consistent across studies. Inconsistent results were found in relation to maternal dimensions, in particular maternal sensitivity [55, 64, 67, 69], as well as to infant dimensions, in particular communicative patterns [58, 62, 69]. A number of factors might support these inconsistencies. Firstly, methods and scoring modalities used to investigate the interaction were very heterogeneous, varying from short video sessions to very long live observations and from microanalytic to global rating scoring systems. Different observational approaches and analyzed dimensions may result in heterogeneous pictures of dyadic exchanges. Secondly, studies were conducted at different infants' ages, albeit within the first year of life. Consequently, observational analyses were quite different across studies and specifically aimed at capturing the most appropriate interactive behaviors at different developmental stages. The last and the most important factor is that all articles included in this review focused on infant populations at high risk or neurologically impaired, but inclusion selection criteria were relatively variable, namely, varying from prematurity only to severe brain lesions. Therefore, not unexpectedly the extent of the interaction impairment was proportional and strictly related to the severity of infant medical risk.

Few studies have reported that early coping maternal behaviors influence later interaction maternal status [53]. Our results show that mothers of older infants demonstrate adaptive interaction strategies based on the impairment level of their infants. In particular, mothers of infants who clearly showed developmental delays as revealed by outcome measurements chose alternative strategies to properly communicate with their infants (i.e., g attention-directing gestures vs questioning) [49, 50]. These findings might indicate a natural maternal attitude to adapt their behaviors based on infant needs over time [45, 49, 53]. Clearly, further investigations are needed in order to extend these results also in view of early interventions aimed at fostering such attitude as early as possible which would be of crucial importance.

In conclusion, results from this work extend previous research which has mainly focused on preterm populations, providing more information relative to early interactions involving infant population with or at high risk for neurological impairments. In fact, while our findings confirm that premature infants displayed behaviors similar to those previously observed in healthy populations, extremely preterm infants and full-term infants with severe illness showed markedly more impaired interactive patterns. Similarly, when maternal behaviors were taken into account, results showed that mothers of high-risk infants were more likely to show altered interactive patterns. However, while the studies reviewed here provided important information, the review did not yield a clear picture of early dyadic interactions in high-risk infant populations. Therefore, further investigation focusing on less heterogeneous populations (e.g., targeting infants with severe perinatal insults only versus controls) and embracing a longitudinal and comprehensive perspective, including, for instance, the systematic evaluation of maternal mental states and their impact on the interaction, are necessary to better characterize the extent to which early parent-infant interactions are impaired following severe perinatal insults. This is an essential step in order to determine the specific impact of addressing the promotion of positive parent-infant interactions as part of early intervention in infants at high neurological risk.

## Figures and Tables

**Table 1:** Characteristics of the included studies.

Study	At high-risk sample	Control sample	Exclusion criteria	Timing of assessment	Method	Main results	Study quality^∗^
Greene et al. [39]	16 PT, with RDS (S-PT) 16 FT, with birth asphyxia (S-FT)	14 PT, healthy (H-PT) 16 FT, healthy (H-FT)	Not defined	At 3 months (CA)	(i) Free play interaction (ii) Video recording: 15 min (iii) At the laboratory (iv) Checklist by Lewis [40]	(i) Infant—look/gaze at mother: H-PT, H-FT > S-PT, S-FT (ii) Mother–vocal responsivity: H-PT, S-PT > H-FT, S-FT (iii) Mother–proximal and kinesthetic stimulation: S-FT > S-PT, H-FT, H-PT (iv) Mother—affective and distal stimulation: S-FT < S-PT, H-FT, H-PT *Other measures*: S-FT, S-PT lower scores in orientation cluster at NBAS	Good

Lasky et al. [41]	40 PT, BW <1500 g and/or requiring mechanical ventilation (PT)	25 FT, healthy (H-FT)	Not defined	At 12 months (CA)	(i) Free interaction in 5 different situations: waiting, physical exam with a nurse present, physical exam with the nurse absent, the nurse return, and blood drawing (ii) Live observation: 5, 2, 2, 2, and 4 min, respectively (iii) At the hospital (iv) Checklist by Lasky et al. [41]	(i) Mother—restrain infant, positioning near infant, looking at the infant, not smiling at infant during blood draw: H-FT > PT *Other measures*: Bayley Mental Developmental Index (MDI) and Psychomotor Developmental Index (PDI): PT < H-FT	Good

Minde et al. [42]	20 PT, BW <1500 g (PT)	20 FT, healthy (H-FT)	No physical malformation	At 1, 2, and 3 months (CA)	-Visit 1 and 2: routine feeding, visit 3: face-to-face play sequence. -Live observation, visits 1-2: duration not reported, visit 3: 10 min -At home -Scoring of infant and maternal behaviors (as in Minde et al. [43])	(i) Feeding 4 weeks: Infant—alert/focused: H-FT>PT Mother—look en face: PT>H-FT Mother—vocalize to others: PT>H-FT Mother—touch: PT<H-FT Mother—smile: PT<H-FT (ii) Feeding 8 weeks: Infant—leg movements: H-FT>PT Mother—vocalize to others: PT>H-FT Mother—vocalize to baby: PT>H-FT Mother—touch: PT<H-FT Mother—smile: PT<H-FT (iii) Play 12 weeks: Infant—head/mouth movements: PT>H-FT Mother—look: PT>H-FT Mother—vocalize to baby: PT>H-FT Mother—smile: PT<H-FT *Other measure*: “Sicker infants” defined according to a morbidity scale by Minde et al. [44] display more behavioral disorganization during feeding, cry more, and have their eyes open.	Good

Landry et al. [45]	20 high-risk (HR) PT with IVH III-IV, RDS, or BPD (HR-PT)	20 low-risk (LR) PT with transient to moderate RDS and/or IVH I-II (LR-PT) 20 FT: healthy full term (H-FT)	Not defined	12 months (CA)	-Free play interaction -Video recording—10 min -At the laboratory -Coding of mothers' attention-directing and infants' exploratory strategies [45]	-Mother—attention-directing behaviors: HR-PT>LR-PT, H-FT; -Mother—questions: H-FT>LR-PT=HR-PT *Other measures*: mothers of HR-PT used questions more often with infants with higher MDI, while they use attention-directing gestures with infants with lower MDI	Good

Farel et al. [46]	37 High-risk (HR) infants with intracranial hemorrhage and/or perinatal asphyxia and/or seizures and/or meningitis and/or BW < 1500 (HR)	37 healthy (H) infants matched for age, sex, and race	Not defined	At 8 months (CA)	-Interactions during a feeding episode, a session in which the mother is asked to teach the child an age-appropriate task and a free play session. -Live observation: 120 min in total -At home -NCAFS, NCATS, and HOME scale (including an interview to the mother) [47, 48]	(i) Dyads—scores at NCAFS and NCATS: HR<H *Other measures*: a strong association between NCAFS and risk status was reported	Poor

Landry et al. [49]	11 PT with IVH III or IV (HR-PT)	16 PT with RDS or IVH I and II (LR-PT) 12 FT, healthy (H-FT)	Other medical complications	At 12 months (CA)	-Toy-centered play interaction -Video recording: 10 min -At the laboratory -Coding of mother's attention-directing and infant's exploratory strategies [45]	-Infant—exploratory play in response to mother's structured strategy: LR-PT>H-FT -Infant—exploratory play: HR-PT<H-FT, and LR-PT -Infant—exploratory play in response to unstructured versus structured strategies: H-FT>LR-PT, HR-PT -Infant—exploratory play in response to structured versus unstructured strategies: LR-PT>H-FT, HR-PT	Fair

Smith et al. [50]	89 PT with BPD, IVH III or IV, and/or PVL (HR-PT)	123 PT with IVH I or II, transient RDS (LR-PT) 128 FT: healthy (H-FT)	Sensory impairments, meningitis, encephalitis, congenital abnormality of the brain	At 6 and 12 months (CA)	-Toy play session and naturalistic observation of daily activity -Live observation—10 min and 60 min -At home -Mixed rating scale and microanalytic coding system [45, 50]	(i) Daily activities and toy play at 6 and 12 months: Mother—interactive behaviors: HR-PT=LR-PT-H-FT *Other measures*: 6 months (i) MDI, daily living, receptive language: FT, LR-PT>HR-PT (ii) Expressive language: HR-PT<H-FT (iii) Positive correlation between mother attention maintaining behavior and infant mental age and receptive language score (during daily activity and toy play): HR-PT, LR-PT>H-FT (iv) Positive correlation between mother attention maintaining behavior and infant expressive language score (during daily living activity): HR-PT, LR-PT>H-FT (v) Positive correlation between mother—attention maintaining behavior and infant expressive language score (during toy play):HR-PT>LR-PT, H-FT	Fair

Schermann-Eizirik et al. [51]	67 PT, GA < 32 was required IC^∗∗^ (VPTIC) 75 PT, GA < 36 was required IC^∗∗^ (PTIC) 66 FT, required IC^∗^^∗^ (FTIC) ^∗∗^IC, intensive care for CPAP or parenteral nutrition o severe asphyxia	70 FT, healthy (H-FT)	Chromosoml abnormalities and severe cerebral malformations.	At 2, 4, and 6 months (CA)	-Interaction during undressing of the infant and face-to-face situation -Video recording—variable time, 3 min -At the laboratory -Mother-infant interaction coding [52]	2,4,6 months: -Mother—interactive behaviors: VPTIC, PTIC=H-FT -Infant—interactive behaviors: VPTIC, PTIC=H-FT -Dyad—positive interaction: VPTIC, PTIC=H-FT 2 months: -Mother—interactive behaviors: FTIC=FT -Infant—interactive behaviors: FTIC=FT 4 months: Mother—sensitivity/involvement: FTIC<FT Infant—interactive behaviors: FTIC<FT Dyad—positive interaction: FTIC<FT (iv) 6 months: Mother—sensitivity/involvement: FTIC<FT Infant—interactive behaviors: FTIC=FT	Good

Davis et al. (2003) [53]	50 PT, BW < 1500 g, with neurobiological risk (defined by NBRS) (HR-PT)	Normative data	No congenital anomalies	Within the 6^th^ month	-Interaction during feeding -Video recording—20 min -At home -NCAFS [47]	(i) Total feeding score: HR-PT<normative data (ii) Mother—interactive behaviors: HR-PT=normative data (iii) Infant—responsivity to caregiver: HR-PT<normative data *Other measures*: mothers who coped better had more responsive children at three months after discharge, according to the Coping Health Inventory for Parents [54]	Fair

Muller-nix et al. [55]	28PT, high-risk (HR) PT, defined by PERI [56] (HR-PT)	19 low-risk (LR) PT, PT, defined by PERI (LR-PT) 25 FT, healthy (H-FT)	Infant malformation, chromosomic abnormalities, foethopaty	At 6 months (CA)	-Mother-child toy-play interaction -Video recording: 10 minutes -Context not specified -CARE index [57]	(i) Mother—sensitivity: HR<LR<FT (ii) Infant—interactive behaviors: HR-PT=LR-PT-H-FT *Other measures*: Mother—posttraumatic stress symptoms: HR-PT>FT More stressed mothers were less sensitive and more controlling in dyadic play	Fair

Schmücker et al. (2005) [58]	79 PT, BW < 1500 g, and/or with IVH, PVL, SGA or required more than 28 days on mechanical ventilation (HR-PT)	35 FT, healthy (H-FT)	Not defined	At 3 months (CA)	-Interaction during diaper change and free play -Video recording—10 minutes -At the laboratory -Microanalytic coding system to rate early mother–child interaction [59]	-Infant-vocalize: HR-PT>H-FT -Infant-vocally responsive: HR-PT>H-FT -Infant—facially responsive: HR-PT<H-FT Mother—facially responsive: HR-PT<H-FT *Other measures*: The higher the neurobiological risk of the infant, the more mothers were judged to lack sensitivity	Fair

Feldman (2006) [60]	17 PT, BW: < 1000 g, GA < 30 ws (HR-PT)	25 PT BW= 1700-1850 g, GA = 34-35 ws (LR-PT) 29 FT: GA >2500 g > 36 ws, (H-FT)	IVH III and IV, asphyxia, metabolic and genetic diseases.	At 3 months (CA)	-Face-to-face interaction -Video recording: 5 min -At home -Scoring with Monadic Phase Manual [61]	-Mother-infant synchrony: H-FT>HR-PT, LR-PT HR-PT=LR-PT -Mother-infant degree of synchrony (coherence): FT>LR-PT>HR-PT -Infant—negative emotionality: HR-PT, LR-PT>H-FT *Other measures*: Biological rhythm analysis revealed that sleep–wake cyclicity, vagal tone, orientation, and arousal modulation are each uniquely predictive of mother–infant synchrony at 3 months	Good

Feldman and Eidelman [62]	18 PT, SGA, BW: <1000 g (SGA< 1000) 28 PT, AGA, BW: <1000 g (AGA< 1000)	22 PT, SGA, BW: >1000 g (SGA > 1000) 52 PT, AGA, BW: >1000 g (AGA > 1000)	IVH IV, asphyxia, metabolic or genetic or syndromic disease, SNC infections	At 3 months (CA)	-Mother-infant interaction -Video recording: 10 min -At home -CIB [63]	Mother-intrusiveness: (SGA<1000)>(SGA>1000)>(AGA<1000), (AGA>1000) Infant—negative engagement: (SGA < 1000) = (SGA > 1000) = (AGA < 1000) = (AGA > 1000) *Other measures*: At 12 months, SGA < 1000 showed poorer cognitive development at MDI. SGA < 1000 scored significantly lower on orientation and motor maturity compared with other groups	Good

Feldman [64]	34 PT, BW < 1500 g; (HR-PT) 21 PT, IUGR, <1500 g (HR-PT)	38 FT: healthy (H-FT)	Maternal and/or paternal depression and anxiety	At 4 months (CA)	-Interaction mother-infant father-infant, triadic interaction -Video recording: 5 min each -At home -Coding Interactive Behavior (CIB) Manual [63]	-Mother—intrusiveness: HR-PT>H-FT -Mother—sensitivity: HR-PT<H-FT -Infant—negative emotionality: HR-PT>H-FT -Dyad—reciprocity: HR-PT<H-FT *Other measures*: (i) Family cohesion: HR-PT<FT -Family rigidity: HR-PT>FT Mother of IUGR infants showed the highest intrusiveness scores and IUGR infants showed the highest negative emotionality. Family also showed the highest rigidity	Good

Korja et al. [65]	30 PT, BW <1500 g, GA: <32 WS (PT)	36 FT: healthy (H-FT)	Major congenital anomalies	At 6 and 12 months (CA)	-Free play mother-infant interaction (toy optional) -Video recording: 5 min -At the laboratory -PC-ERA [66]	-6 months: Infant—interactive behaviors: PT=H-FT Mother—interactive behaviors: PT=H-FT -12 months: Infant—quality of play and attention: PT<H-FT Infant-sober and withdrawn: PT<H-FT Mother—interactive behaviors: PT=H-FT *Other measures*: duration of holding at 5 months (CA) was positively associated with the good quality of mother–infant interaction at 6 and 12 months in PTPTs cried (combined fussing and crying) more often and were held more than H-FT	Good

Agostini et al. [67]	29 PT, BW: <1000 g (HR-PT) 40 PT, BW: <1500 g (PT)	80 FT healthy (H-FT)	Infant chromosomal abnormalities, CP, malformations and foetopathy	At 3 months (CA)	-Face-to-face interaction -Video recording: 5 min -At the laboratory -Global rating scale (GRS) [68]	-Mother—sensitivity: HR-PT=PT=H-FT -Mother—intrusiveness: HR-PT>H-FT -Mother—remoteness: PT, HR-PT<H-FT -Infant—interactive behaviors: HR-PT=PT=H-FT *Other measures*: In H-FT mothers, higher degree of remoteness was associated to the presence of depressive symptoms	Good

Neri et al. [69]	32 PT, BW: <1000 g (HR-PT) 45 PT, BW <1500 g (PT)	20 FT, healthy (H-FT)	Infant chromosomal abnormalities, cerebral palsy, malformations and foetopathy	At 3 months (CA)	-Face-to-face interaction -Video recording: 5 min -At the laboratory -Global rating scale (GRS) [68]	-Mother—sensitivity: PT>H-FT -Mother—intrusiveness: HR-PT>H-FT -Mother—remoteness: HR-PT<H-FT -Infant—communicative dimension: PT>FT *Other measures*: Mother—signs of depression: HR-PT, PT>H-FT	Good

Sansavini et al., 2015 [70]	20 PT: GA < 28 ws (HR-PT)	20 FT: >37 ws (H-FT)	Major cerebral damage, PVL, IVH > II grade, hydrocephalus	At 12 months (CA)	-Mother-child toy-play interaction -Video recording: 10 minutes -At the laboratory -R-RCS for dyadic coregulation and Lunkenheimer's coding system for coding affective intensity [71, 72]	-Dyad—frequency of symmetric coregulation patterns: HR-PT<H-FT -Dyad—frequency of unilateral coregulation patterns: HR-PT>H-FT -Infant—emotional Involvement: HR-PT<H-FT -Mother—frequency of high positive affective intensity: HR-PT<H-FT Infant—frequency of neutral affective intensity: HR-PT>H-FT Infant—frequency of high positive affective intensity: HR-PT<H-FT Infant—frequency of low positive affective intensity: HR-PT>H-FT Infant—duration of neutral affective intensity: HR-PT>H-FT Infant—duration of high positive affective intensity: HR-PT<H-FT Infant—duration of low positive affective intensity: HR-PT<H-FT Infant—duration of neutral affective intensity: HR-PT>H-FT Infant—duration of low negative affective intensity: HR-PT>H-FT	Fair

AGA = appropriate for gestational age; BPD = bronchopulmonary dysplasia; BW = birth weight; CA = corrected age; CIB = coding interactive behavior; FT = full-term infant; GA = gestational age; H-FT = healthy full term; H-PT = healthy preterm; HOME = Home Observation Measurement of the Environment; HR-PT = high-risk preterm; IUGR = intrauterine growth retardation = IVH: intraventricular hemorrhage; LR-PT = low-risk preterm; MDI = Bayley Mental Developmental Index; NBAS = Neonatal Behavioral Assessment Scale; NBRS = Neurobiological Risk Score; NCAFS = Nursing Child Assessment Feeding Scale; NCATS = Nursing Child Assessment Teaching Scale; PC-ERA = Parent-Child Early Relational Assessment; PDI = Bayley Psychomotor Developmental Index; PT = preterm infant; PTIC = preterm who required intensive care; PVL = periventricular leukomalacia; RDS = respiratory distress syndrome; S-FT = sick full term; S-PT = sick preterm; SGA = small for gestational age; VPTIC = very preterm who required intensive care. ^∗^Assessed through National Heart, Lung, and Blood Institute (NHLBI) Quality Assessment Tool for Case-Control Studies [38].

**Table 2:** Assessment and scoring scales used in the studies.

Assessment and scoring scales	Description	Reference	Studies
*Global scales*			
Global rating scales (GRS)	Assessment of the quality of mother-infant interaction. Maternal behavior is rated on 4 dimensions: sensitivity, intrusiveness, remoteness, and signs of depression. Infant behavior is rated on 3 dimensions: communicative, inert, and distressed. One dimension assesses the quality of the overall interaction between mother and infant. A 5-point Likert-type scale is used to rate each dimension, with 1 being the poorest and 5 being the optimal rating.	Gunning et al. Murray et al. [15, 73]	Agostini et al. [67]; Neri et al. [69]
NCAST Feeding (NCAFS) and Teaching (NCAST) PCI Scales	The NCAST-PCI evaluates 149 items related to maternal and infant behaviors. It comprises two scales: NCAFS and NCATS. Infant and parent items are coded as yes or no; items are then added to provide a total score. Each scale includes 4 subscales, measuring maternal behaviors and 2 subscales, measuring infant's behaviors. Maternal subscales are sensitivity to cues, responsivity to child's distress, social-emotional growth fostering, and cognitive growth fostering. Infant subscales are clarity of cues and responsiveness to parent.	Barnard et al. [47]	Davis et al. [53] Farel et al. [46]
Coding Interactive Behavior (CIB)	Global rating system of parent-child interaction in different play or interaction situations, including 42 codes: 21 for parents, 16 for infants, and 5 for dyads. Each score is rated with a Likert-type scale, where 1 corresponds to the poorest and 5 to the optimal rating. Five composite scales are included: maternal sensitivity, maternal intrusiveness, child's social involvement, and dyadic reciprocity.	Feldman [63]	Feldman and Eidelman [62]; Feldman [64]
Parent-Child Early Relational Assessment (PC-ERA)	Semistructured assessment to evaluate affective and behavioral quality of parent–infant interaction during 4 situations: feeding, administration of a structured task, free play, and a separation-reunion task. Three parental subscales (29 items) are coded: positive affective involvement and verbalization, negative affect and behaviors, and intrusiveness, insensitivity, and inconsistency. Three Infant subscales (28 items) are coded: positive affect, social and communicative competence; quality of play, interest and attentional skills; dysregulation and irritability. Two dyadic subscales (8 items) are coded: mutual enjoyment and reciprocity, tension and disorganization. A 5-point Likert-type scale is used to rate each item.	Clark [66]	Korja et al. [65]
CARE-Index	Assessment of the quality of adult-infant interaction. Three adult behaviors are scored: sensitivity, control, and unresponsiveness. Four infant behaviors are scored: cooperativeness, compulsiveness, difficultness, and passivity. The scores range from 0 to 14, with 0 score being the worst score.	http://www.patcrittenden.com, Crittenden 1979-2004 [57]	Muller-nix et al. [55]
HOME	Inventory designed to identify the presence of risk for developmental delay due to lacking of appropriate quantity and quality of stimulation from home environment. Forty-five binary items, organized in six subscales, are scored using a combination of semistructured mother interview relative to children routine activities, observation of mother-infant interaction during play and interview and assessment of kinds of play materials available to the child. Six subscales are coded: emotional and verbal maternal responsivity, maternal avoidance of restriction and punishment, maternal involvement with the child, organization of the environment, provision of appropriate play materials, and variety in daily stimulation.	Bradley and Cadwell [48]	Farel et al., [46]
*Microanalytic*			
Coding system of Minde (1980)	Microanalytic system recording the occurrence of the 10 maternal and 11 infant behaviors. Infant behaviors: arm, head, leg, hand to mouth; eyes open; scan; grimace; cry; vocalize; smile; yawn. Maternal behaviors: look; look *en face*; verbalize to baby and to others; instrumental and noninstrumental touch; hold; feed; smile; standing further than 1 meter away from the baby.	Minde et al. [43]	Minde et al. [42]
Microanalytic coding system to rate early mother–child Interaction by Jorg (1994)	Microanalytic system which rates interactive behaviors at fixed time intervals of 1, 15, and 30 seconds. In particular, maternal behaviors rated per second are direction of gaze, vocalization, facial expression, content of interaction, and proximity; infant behaviors rated per second are direction of gaze, vocalization, and facial expression; joint mother-child behaviors rated every 15-30 seconds are appropriateness of stimulation, maternal responsiveness, and child responsiveness.	Jorg et al. [59]	Schmücker et al. [58]
Coding system of Landry (1986)	This coding system is based on the recording of the occurrence of mothers' attention-directing strategies and infants' responses. The variables scored are maternal attention-directing attempt, verbal technique-question, verbal technique-imperative, verbal technique-attention verbs, nonverbal techniques attention directing-gesture, nonverbal techniques attention directing-demonstrate, nonverbal techniques attention directing-give, initial focus of attention-maintain, initial focus of attention-introducing, initial focus of attention-redirecting, infant response-no response, infant response-look, infant response-manipulate.	Landry [45]	Landry [45]; Landry [49]
Monadic Phase Manual	Coding system in which the stream of affective behavior of each partner is coded using 6 expressive modalities for the parent, which are vocalization, direction of gaze, head orientation, facial expression, body position, and specific handling of the infant and 5 for the infant which are vocalization, direction of gaze, head orientation, and facial expression. Combination of expressive modalities, checked second by second, is transformed in one of the following seven adult monadic phases: avoid, avert, monitor, elicit, set, play, and talk. Six infant monadic phases are also coded: avoid, avert, monitor, set, play, and talk.	Tronick et al. [61] Feldman [74]	Feldman [60]
Revised relational coding system (R-RCS)	This coding scale assesses dyadic coregulation based on 5 patterns: symmetrical, asymmetrical, unilateral, disruptive, and unengaged. One additional pattern, no code, can be used for missing information.	Fogel et al. [71]	Sansavini [70]
Lunknenheimer's coding system	This scale codes parent and infant positive and negative affective intensity in 30 s intervals. An ordinal 3-point scale (non, low, high) is used to code affective behaviors based on a combination of voice tone, facial expression, eye contact, and body language.	Lunknenheimer et al. [71]	Sansavini [70]
*Checklist*			
Checklist by Lewis	Checklist sheet for recoding 13 infant and 12 maternal behaviors. Behavior are coded within 10-second periods: occurrence, initiation, or response. Two principal types of summary variables are computed from discrete infant and maternal behaviors: general behavior and responsivity. Maternal general behaviors: frequency of general stimulation; frequency of proximal stimulation, frequency of distal stimulation, frequency of kinesthetic stimulation, frequency of positive affect expression, and frequency of vocal stimulation. Infant general behaviors: frequency of fret/cry, frequency of vocalization, and frequency of look/gaze at mother. Maternal responsivity: proportion of general responsivity, proportion of proximal responsivity, proportion of distal responsivity, and proportion of vocal responsivity. Infant responsivity: proportion of general responsivity.	Lewis et al. [40]	Greene et al. (1983) [39]
Checklist by Lasky et al., 1984	Observational method based on rating the presence of maternal and infant behaviors in 5 different situations. 10 behaviors initiated by the infant and 12 behaviors initiated by the mother are checked.	Lasky et al. [41]	Lasky et al. [41]
Checklist by Bohlin et al., 1989	Observational method based on a 5-point scale (higher score indicating higher frequency or better performance) rating of maternal, infant, and dyadic items. Maternal items are grouped into three variables: sensitivity, intrusiveness, and involvement. Infant items are grouped into two variables: infant interactive behaviors. The dyadic variable corresponds to a global evaluation of quality of positive interaction.	Bohlin et al., [52]	Schermann-Eizirik et al. [51]
*Other*			
Mixed rating scale and microanalytic coding system	Five-point rating scale to code a composite measure labeled “warm sensitivity” which comprises three maternal behaviors: positive affect, warm concern/acceptance, and flexibility/responsiveness combined to a microanalytic coding scheme developed to quantify maternal attention-directing events defined as verbal and nonverbal behaviors (frequency of events is considered for analysis).	Landry et al., [45] Smith et al., [50]	Smith et al. [50]
